# Measurement error adjustment in essential fatty acid intake from a food frequency questionnaire: alternative approaches and methods

**DOI:** 10.1186/1471-2288-7-41

**Published:** 2007-09-14

**Authors:** May A Beydoun, Jay S Kaufman, Joseph Ibrahim, Jessie A Satia, Gerardo Heiss

**Affiliations:** 1 Center for Human Nutrition, Department of International Health, Bloomberg School of Public Health, Johns Hopkins University, Baltimore, MD, USA; 2 Department of Epidemiology, School of Public Health, University of North Carolina at Chapel Hill, Chapel Hill, NC, USA; 3 Department of Biostatistics, School of Public Health, University of North Carolina at Chapel Hill, Chapel Hill, NC, USA; 4 Department of Nutrition, School of Public Health, University of North Carolina at Chapel Hill, Chapel Hill, NC, USA

## Abstract

**Background:**

We aimed at assessing the degree of measurement error in essential fatty acid intakes from a food frequency questionnaire and the impact of correcting for such an error on precision and bias of odds ratios in logistic models. To assess these impacts, and for illustrative purposes, alternative approaches and methods were used with the binary outcome of cognitive decline in verbal fluency.

**Methods:**

Using the Atherosclerosis Risk in Communities (ARIC) study, we conducted a sensitivity analysis. The error-prone exposure – visit 1 fatty acid intake (1987–89) – was available for 7,814 subjects 50 years or older at baseline with complete data on cognitive decline between visits 2 (1990–92) and 4 (1996–98). Our binary outcome of interest was clinically significant decline in verbal fluency. Point estimates and 95% confidence intervals were compared between naïve and measurement-error adjusted odds ratios of decline with every SD increase in fatty acid intake as % of energy. Two approaches were explored for adjustment: (A) External validation against biomarkers (plasma fatty acids in cholesteryl esters and phospholipids) and (B) Internal repeat measurements at visits 2 and 3. The main difference between the two is that Approach B makes a stronger assumption regarding lack of error correlations in the structural model. Additionally, we compared results from regression calibration (RCAL) to those from simulation extrapolation (SIMEX). Finally, using structural equations modeling, we estimated attenuation factors associated with each dietary exposure to assess degree of measurement error in a bivariate scenario for regression calibration of logistic regression model.

**Results and conclusion:**

Attenuation factors for Approach A were smaller than B, suggesting a larger amount of measurement error in the dietary exposure. Replicate measures (Approach B) unlike concentration biomarkers (Approach A) may lead to imprecise odds ratios due to larger standard errors. Using SIMEX rather than RCAL models tends to preserve precision of odds ratios. We found in many cases that bias in naïve odds ratios was towards the null. RCAL tended to correct for a larger amount of effect bias than SIMEX, particularly for Approach A.

## Background

Food frequency questionnaires (FFQ) have been historically used in large epidemiological studies to assess usual dietary intake of specific nutrients and food groups. They have been repeatedly validated against another dietary assessment method assumed to be more accurate such as multiple 24-hour recalls or food records of food intake [1-3]. The problem inherent in this approach is that the same factors that affect these reference methods (*R*) may also affect the FFQ-based assessments (*Q*), which include over or under-reporting biases of subjects with certain socio-demographic or health-related characteristics. This problem would make it impossible to presume independent random errors in the two methods, which in turn leads to over-estimation of the correlation between the reference method and the FFQ [4]. Hence, to consider a method a gold standard, it should ideally reflect the true values of what we are trying to measure. In nutritional epidemiology, very few biomarkers can be considered as gold standards and are often very expensive to carry out even on relatively small samples. Alloyed gold standards are used instead which are considered to be accurate depictions of the truth, being unbiased in expectation. Moreover, any error associated with them can be assumed to be random and independent of the true unknown value of intake. Compared to self-reports (e.g. multiple 24 hr. recalls), biomarkers are alloyed gold standards that can additionally be assumed to have independent measurement errors from those of the test measure itself (i.e. FFQ). Such restriction on measurement error associations makes biomarkers a desirable target for validation studies. A number of epidemiologic studies however, consider repeat measures of the FFQ as alternative means to correct for measurement error, given that biomarkers have also some drawbacks including their associated random and systematic error components. In fact, a previous study favored this method over external validation in terms of preserving the precision of estimates [5].

The present study aims first at assessing the degree of measurement error in the intake of essential fatty acids using a food frequency questionnaire, by estimating attenuation factors for each exposure. Second, we studied the impact of correcting for such measurement error on bias and precision of odds ratios from a multivariate logistic regression model. For illustrative purposes, we examined the effect of dietary essential fatty acids on clinically significant cognitive decline among older adults, controlling for other potential confounders. For both objectives, two alternative approaches were used: (A) *External validation against two biomarkers *measured at visit 1 among a subset of the Atherosclerosis Risk in Communities (ARIC) study. These concentration biomarkers are the percent of each fatty acid or group of fatty acids out of total fatty acid concentration in the cholesteryl ester (*M*) and phospholipids (*N*) fractions of plasma which were previously shown to be linearly related to dietary intake as assessed by more reliable reference methods such as multiple 24-hour recalls, food records or diet history [6-8]. In fact, one study conducted in UK (Whitehall II) showed that correlation between 7-day food records estimated fatty acids and cholesteryl ester concentration was 0.38 for EPA (one major n-3 highly unsaturated fatty acid), 0.67 for linoleic acid and 0.56 for all polyunsaturated fatty acids. The other studies came to a similar conclusion. Hence, despite the issue of transportability, it can be concluded that M and N are moderately correlated with reference measures of dietary intake (B) *Internal repeat measurement of the FFQ *values at visits 2 and 3. While the former approach eliminates the possibility of correlated errors between test and reference method (one being a self-report and the other a biomarker), the second approach is often more readily available in many large cohort studies. In addition, for the second objective, we contrasted regression calibration with another method known as simulation extrapolation or SIMEX. Previous research indicated that dietary n-3 fatty acids were suggestive of a protective effect against cognitive aging [9-13]. Other studies of concentration biomarkers for n-3 fatty acids indicated a similar association [14-18]. However, a number of other reports could not replicate these findings [19,20]. An evidence-based report suggested a need to look for the effect of *n*-3 fatty acids on cognitive decline and to define exposure in terms of absolute value of medium chain and long chain fatty acids, as well as the ratio between n-3 and n-6 fatty acids in diet and plasma [21].

Findings from our study and its overall methodology may be used in subsequent analyses to adjust for measurement error in primary regression linking intake of essential fatty acids with various disease outcomes.

## Results

### Fatty Acid Exposure Distribution

Table 1 presents the distribution of fatty acid groups and ratios for the main study as well as the sub-samples with complete biomarker and repeat measure data. Fatty acid groups are expressed as % of total energy for *Q*_1_, *Q*_2 _and *Q*_3_. For *M *and *N*, they were expressed as % of total fatty acids in the plasma fraction. A decreasing trend in reported consumption of groups *6P *and *6 *was noted. Similarly, the ratio of reported 3H/6H declined almost linearly over visits 1, 2 and 3. Standard deviations of *Q*_1 _are instrumental in interpreting results for logistic regression model analyses.

**Table 1:** Distribution of fatty acid groups and ratios Q_1_, M, N, Q_2 _and Q_3 _: Mean ± SD; ARIC (1987–1995)^1^

	**Q_1_**	**M**	**N**	**Q_2_**	**Q_3_**
			
	(n = 7,814)	(n = 2,251)		(n = 634)	
**Fatty acid groups and ratios *j*^2^**					
**6p**	4.43 ± 1.43	55.22 ± 4.46	22.03 ± 2.59	4.29 ± 1.40	4.19 ± 1.36
**3p**	0.41 ± 0.09	0.41 ± 0.10	0.14 ± 0.05	0.40 ± 0.09	0.41 ± 0.10
**6H**	0.08 ± 0.03	9.11 ± 1.70	15.80 ± 2.10	0.07 ± 0.03	0.07 ± 0.03
**3H**	0.18 ± 0.16	1.01 ± 0.39	3.44 ± 1.05	0.17 ± 0.15	0.16 ± 0.15
**6**	4.50 ± 1.43	63.31 ± 4.01	37.73 ± 1.78	4.37 ± 1.41	4.27 ± 1.36
**3**	0.60 ± 0.19	1.42 ± 0.43	3.59 ± 1.05	0.57 ± 0.18	0.57 ± 0.18
**3p/6p**	0.10 ± 0.04	0.01 ± 0.00	0.01 ± 0.00	0.10 ± 0.02	0.11 ± 0.05
**3H/6H**	2.27 ± 1.87	0.11 ± 0.05	0.22 ± 0.08	2.22 ± 1.71	2.12 ± 1.69
**3/6**	0.15 ± 0.07	0.02 ± 0.01	0.09 ± 0.03	0.14 ± 0.07	0.15 ± 0.07

^1 ^Q_1_: Food frequency questionnaire measurement at visit 1 of fatty acid group intake as % of energy intake or ratio of n-3 to n-6 groups. M: biomarker of fatty acid intake in cholesteryl ester fraction of plasma; N: biomarker of fatty acid intake in phospholipid fraction of plasma; Q_2_: Repeat of Q_1 _measured at visit 2 among a subset of the cohort; Q_3_: Repeat of Q_1 _measured at visit 3 among the surviving baseline cohort. ^2^**(3P) **n-3 C_18 _polyunsaturated fatty acids: 18:3+18:4n-3 **(6P) **n-6 C_18 _polyunsaturated fatty acids: 18:2+18:3n-6 **(3H) **n-3 C_20 _and C_22 _highly unsaturated fatty acids (HUFAs): 20:5+22:5+22:6n-3 and **(6H) **n-6 HUFAs: 20:3+20:4+22:4+22:5n-6. Sums of fatty acid intake as percent of energy included (3) = (3P)+(3H) and (6) = (6P)+(6H). Ratios of interest: **3P/6P**, **3H/6H **and (3P+3H)/(6P+6H) also denoted as **3/6**. *Notation*: The chemical structure of each fatty acid is as follows: "Total number of carbon atoms" : "# of double bonds" n-"carbon number with first double bond starting from the methyl end.

### Measurement error in fatty acid exposures: attenuation factors estimation with two alternative approaches

Table 2 presents the attenuation factor estimates from the measurement error models using Approaches A (external validation with biomarkers *M/N*) and B (Internal repeat measurements *Q*_2_*/Q*_3_). Our findings suggested that attenuation of bivariate effects was more pronounced for approach A with factors ranging between 0.046 for 6H and 0.426 for 3H/6H. As for Approach B, attenuation factors ranged between 0.456 for 3P and 0.655 for 3H/6H and 3/6. Further, a sensitivity analysis with assumed naïve odds ratios of two for all *j *variables (Log_e_(OR_naive_) = 0.693 with SE = 0.10) was conducted to get a feel of the magnitude in attenuation in a measure of association that is commonly used in epidemiologic studies. Equations **4.1–4.4 (Methods section) **were used for this purpose. In many cases, approach A yielded corrected odds ratios that were outside the reliable range (The two odds ratios differed by more than 10-folds) while approach B gave adjusted odds ratios within a reliable range. However, further sensitivity analysis with other values of the naïve estimate of the odds ratio indicated that a value between 1.0 and 1.5 gave more reliable corrected estimates compared to values of 1.5 or higher, particularly for approach A. It is worth noting that these computations are only applicable to bivariate models with one error prone variable.

**Table 2:** Attenuation factor estimates (with standard error) from the two approaches and regression calibrated odds ratio^2^: Bivariate logistic regression model; ARIC (1987–1998)^1^

Fatty acid groups and ratios *j*^3^	External validation with biomarkers (**M/N**)			Internal repeat measurements (**Q_2_/Q_3_**)		
	
	Attenuation factor λ^j MathType@MTEF@5@5@+=feaafiart1ev1aqatCvAUfKttLearuWrP9MDH5MBPbIqV92AaeXatLxBI9gBaebbnrfifHhDYfgasaacH8akY=wiFfYdH8Gipec8Eeeu0xXdbba9frFj0=OqFfea0dXdd9vqai=hGuQ8kuc9pgc9s8qqaq=dirpe0xb9q8qiLsFr0=vr0=vr0dc8meaabaqaciaacaGaaeqabaqabeGadaaakeaaiiGacuWF7oaBgaqcamaaBaaaleaacqWGQbGAaeqaaaaa@3001@	Standard error (SE λ^j MathType@MTEF@5@5@+=feaafiart1ev1aqatCvAUfKttLearuWrP9MDH5MBPbIqV92AaeXatLxBI9gBaebbnrfifHhDYfgasaacH8akY=wiFfYdH8Gipec8Eeeu0xXdbba9frFj0=OqFfea0dXdd9vqai=hGuQ8kuc9pgc9s8qqaq=dirpe0xb9q8qiLsFr0=vr0=vr0dc8meaabaqaciaacaGaaeqabaqabeGadaaakeaaiiGacuWF7oaBgaqcamaaBaaaleaacqWGQbGAaeqaaaaa@3001@)	RCAL odds ratio (95% CI)^2^	Attenuation factor λ^j MathType@MTEF@5@5@+=feaafiart1ev1aqatCvAUfKttLearuWrP9MDH5MBPbIqV92AaeXatLxBI9gBaebbnrfifHhDYfgasaacH8akY=wiFfYdH8Gipec8Eeeu0xXdbba9frFj0=OqFfea0dXdd9vqai=hGuQ8kuc9pgc9s8qqaq=dirpe0xb9q8qiLsFr0=vr0=vr0dc8meaabaqaciaacaGaaeqabaqabeGadaaakeaaiiGacuWF7oaBgaqcamaaBaaaleaacqWGQbGAaeqaaaaa@3001@	Standard error (SE λ^j MathType@MTEF@5@5@+=feaafiart1ev1aqatCvAUfKttLearuWrP9MDH5MBPbIqV92AaeXatLxBI9gBaebbnrfifHhDYfgasaacH8akY=wiFfYdH8Gipec8Eeeu0xXdbba9frFj0=OqFfea0dXdd9vqai=hGuQ8kuc9pgc9s8qqaq=dirpe0xb9q8qiLsFr0=vr0=vr0dc8meaabaqaciaacaGaaeqabaqabeGadaaakeaaiiGacuWF7oaBgaqcamaaBaaaleaacqWGQbGAaeqaaaaa@3001@)	RCAL odds ratio (95% CI)^2^
				
**6p**	0.230	(0.020)	20.3 (11.8, 35.0)	0.605	(0.031)	3.1 (2.7, 3.7)
**3p**	0.090	(0.022)	>100	0.456	(0.033)	4.6 (3.5, 5.9)
**6H**	0.046	(0.028)	>100	0.653	(0.027)	2.9 (2.6, 3.2)
**3H**	0.408	(0.020)	5.5 (4.5, 6.6)	0.717	(0.025)	2.6 (2.4, 2.9)
**6**	0.269	(0.020)	13.1 (8.8, 19.7)	0.603	(0.031)	3.2 (2.7, 3.7)
**3**	0.353	(0.021)	7.1 (5.5, 9.2)	0.632	(0.027)	3.0 (2.6, 3.4)
**3p/6p**	0.251	(0.021)	15.8 (9.8, 25.6)	0.588	(0.031)	3.2 (2.8, 3.8)
**3H/6H**	0.426	(0.020)	5.1 (4.3, 6,1)	0.655	(0.028)	2.9 (2.5, 3.2)
**3/6**	0.401	(0.021)	5.6 (4.6, 6.9)	0.655	(0.024)	2.9 (2.6, 3.2)

^1 ^Q_1_: Food frequency questionnaire measurement at visit 1 of fatty acid group intake as % of energy intake or ratio of n-3 to n-6 groups. M: biomarker of fatty acid intake in cholesteryl ester fraction of plasma; N: biomarker of fatty acid intake in phospholipids fraction of plasma; Q_2_: Repeat of Q_1 _measured at visit 2 among a subset of the cohort; Q_3_: Repeat of Q_1 _measured at visit 3 among the surviving baseline cohort. RCAL: regression calibrated estimate.

^2 ^Naïve odds ratio assumed to be equal to 2.00 (1.64, 2.43), with Log_e_(odds ratio_naive_) = *β*_naive _= 0.693 with a SE(*β*_naive_) = 0.100, in the following bivariate model: E(Y|Qj)=β^0+β^(naïve, j)Qj
 MathType@MTEF@5@5@+=feaafiart1ev1aaatCvAUfKttLearuWrP9MDH5MBPbIqV92AaeXatLxBI9gBaebbnrfifHhDYfgasaacH8akY=wiFfYdH8Gipec8Eeeu0xXdbba9frFj0=OqFfea0dXdd9vqai=hGuQ8kuc9pgc9s8qqaq=dirpe0xb9q8qiLsFr0=vr0=vr0dc8meaabaqaciaacaGaaeqabaqabeGadaaakeaacqqGfbqrcqqGOaakcqWGzbqwcqqG8baFcqWGrbqudaWgaaWcbaGaemOAaOgabeaakiabbMcaPGGaaiab=1da9GGaciqb+j7aIzaajaWaaSbaaSqaaiabbcdaWaqabaGccqWFRaWkcuGFYoGygaqcamaaBaaaleaacqqGOaakcqqGUbGBcqqGHbqycqqGVdW7cqqG2bGDcqqGLbqzcqqGSaalcqqGGaaicqqGQbGAcqqGPaqkaeqaaOGaemyuae1aaSbaaSqaaiabdQgaQbqabaaaaa@4AB8@. The odds ratio is for any binary outcome (0,1) vs. fatty acid exposure expressed as a z-score (increase in odds of outcome for each 1 SD in fatty acid exposure). Refer to Eq. 4.1–4.4.

^3 ^See table 2.

### Measurement error adjustment in multivariate logistic models: alternative approaches

**Figure 1:**
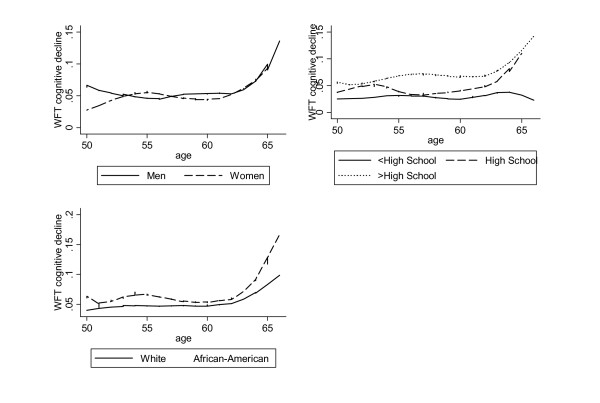
Locally Weighted regression (LOWESS)^1 ^of clinically significant decline in Word Fluency Test (WFT) by age: stratified by sex, education and ethnicity; ARIC 1987–1998. ^1^LOWESS smoother with bandwidth of 0.50.

Figure 1. shows a LOWESS smoothed representation of the behavior of clinically significant cognitive decline in verbal fluency between visits 2 and 4 in our study population (n = 7,814) by age, stratified by sex, education and ethnicity [22]. In general, cognitive decline was shown to affect a greater proportion of individuals for subjects whose baseline age was 60 years or more, and that was particularly true for those with education exceeding high school and among African-Americans. The same patterns were observed when continuous cognitive change was considered (Visit 4 – Visit 2).

As an application to multivariate logistic models, we conducted further analysis using available data on cognitive decline and essential fatty acids and controlling for relevant confounders (Equations 4 and 5). Table 3 presents naïve and corrected odds ratios for each fatty acid group/ratio in relation to decline in WFT using two approaches (external validation and internal repeat measurements) as well as two measurement error correction methods (RCAL and SIMEX). In general, the results showed that RCAL tended to yield wider confidence intervals as compared to SIMEX when contrasting confidence limit ratios, particularly when the naïve estimate was close to the null value of 1. The point estimate indicated bias towards the null value of 1, when comparing the corrected to the naïve for 28 out of the 36 models that were ran. Most of the 8 associations that did not fit this finding showed bias through the null and had naïve odds ratios very close to the null value of 1.00. On average, the absolute bias that is corrected in odds ratios for each approach/method dyad was as follows: Approach A/RCAL (|Δ*bias*| = 0.12); Approach A/SIMEX (|Δ*bias*| = 0.06); Approach B/RCAL (|Δ*bias*| = 0.08); Approach B/SIMEX (|Δ*bias*| = 0.06). Hence, in general, RCAL corrected for more bias than SIMEX did and this was more obvious for Approach A.

**Table 3:** Naïve and corrected odds ratios for each fatty acid group/ratio and decline in Word Fluency Test (WFT)^3 ^using two approaches and RCAL/SIMEX methods: Change in estimate (Δbias) and precision (Δprecision) compared to the naïve estimates; ARIC (1987–1998)^1^

	Odds Ratios (95% Confidence Intervals)									
	
Fatty acid groups and ratios *j*^2^	Naïve (Q_1 _= T)		External validation with biomarkers (**M/N**) (Approach A)				Internal repeat measurements (**Q_2_/Q_3_**) (Approach B)			
			RCAL		SIMEX		RCAL		SIMEX	
**6p**	1.03	(0.93, 1.14)	0.98	(0.76, 1.25)	0.98	(0.83, 1.16)	1.04	(0.75, 1.21)	0.97	(0.80,1.18)
Δbias^4^	__	__	0.05	c	0.05	c	-0.01	a	0.06	c
Δprecision^5^	__	__	1.34		1.14		1.32		1.33	
**3p**	1.01	(0.92, 1.12)	1.02	(0.77, 1.36)	1.01	(0.86, 1.17)	1.22	(0.90, 1.64)	1.15	(0.96,1.39)
Δbias	__	__	-0.01	a	0.00		-0.21	a	-0.14	a
Δprecision	__	__	1.45		1.12		1.50		1.19	
**6H**	1.18	(1.06, 1.31)*	1.63	(1.26, 2.14)*	1.34	(1.14, 1.56)*	1.19	(0.48, 2.91)	1.20	(0.56,2.56)
Δbias	__	__	-0.45	a	-0.16	a	-0.01	a	-0.02	a
Δprecision	__	__	1.37		1.11		4.91		3.70	
**3H**	0.85	(0.75, 0.96)*	0.73	(0.58, 0.90)*	0.80	(0.66, 0.96)*	0.80	(0.62, 1.04)	0.83	(0.68,1.02)
Δbias	__	__	0.12	a	0.05	a	0.05	a	0.02	a
Δprecision	__	__	1.21		1.14		1.31		1.17	
**6**	1.03	(0.97, 1.14)	1.08	(0.82, 1.41)	1.04	(0.89, 1.22)	0.96	(0.76, 1.21)	0.96	(0.81,1.15)
Δbias	__	__	-0.05	a	-0.01	a	0.07	c	0.07	c
Δprecision	__	__	1.46		1.17		1.35		1.21	
**3**	0.94	(0.85, 1.05)	0.71	(0.56, 0.90)*	0.80	(0.65, 0.97)*	0.85	(0.62, 1.17)	0.90	(0.73,1.11)
Δbias	__	__	0.23	a	0.14	a	0.09	a	0.04	a
Δprecision	__	__	1.30		1.21		1.53		1.23	
**3p/6p**	1.01	(0.91, 1.11)	1.03	(0.81, 1.30)	1.03	(0.88, 1.21)	1.21	(0.95, 1.53)	1.14	(0.95,1.36)
Δbias	__	__	-0.02	a	-0.02	a	-0.20	a	-0.13	a
Δprecision	__	__	1.32		1.13		1.32		1.17	
**3H/6H**	0.86	(0.76, 0.97)*	0.77	(0.64, 0.93)*	0.79	(0.65, 0.96)*	0.84	(0.65, 1.10)	0.87	(0.70,1.08)
Δbias	__	__	0.09	a	0.07	a	0.02	a	-0.01	b
Δprecision	__	__	1.14		1.16		1.33		1.21	
**3/6**	0.95	(0.85, 1.06)	0.91	(0.76, 1.10)	0.92	(0.78, 1.08)	1.04	(0.85, 1.27)	1.04	(0.87,1.23)
Δbias	__	__	0.04	a	0.03	a	-0.09	c	-0.09	c
Δprecision	__	__	1.16		1.11		1.20		1.13	

^1 ^Q_1_: Food frequency questionnaire measurement at visit 1 of fatty acid group intake as % of energy intake or ratio of n-3 to n-6 groups. M: biomarker of fatty acid intake in cholesteryl ester fraction of plasma; N: biomarker of fatty acid intake in phospholipid fraction of plasma; Q_2_: Repeat of Q_1 _measured at visit 2 among a subset of the cohort; Q_3_: Repeat of Q_1 _measured at visit 3 among the surviving baseline cohort; WFT: Word Fluency Test; RCAL: Regression Calibration; SIMEX: Simulation Extrapolation.

^2 ^See table 3.

^3 ^Each exposure is entered into the logistic regression model as a z-score and the outcome is binary with 1: decline and 0: no decline based on the reliable change index (RCI) criterion. RCI < -1.645 constitutes clinically significant decline between the two ARIC visits 2 and 4, which were separated by 6 years. The odds ratio is interpreted as increase in risk of cognitive decline with each 1 SD increase in the fatty acid exposure.

^4 ^Δbias = OR_naive _- OR_corrected_. a: naïve estimate is biased towards the null; b: naïve estimate is biased away from the null; c: naïve estimate is biased through the null. The null value for an odds ratio is 1.00.

^5 ^Δprecision = CLR_corrected_/CLR_naive_. CLR or Confidence Limit Ratio is the ratio of the upper 95% confidence limit over the lower one.

For internal repeat measurements (Approach B), results indicated an appreciable loss in precision, given that replicate *Q*_2 _was measured for only 657 subjects out of the 7,814 who were eligible. Comparing the two approaches, many of the unexpected results with bias through or away from the null occurred in Approach B. In addition, in terms of precision, approach A yielded an average for ratios of confidence limit ratios (CLRs) of 1.22 (1.41 for RCAL and 1.14 for SIMEX), compared to 1.62 (1.75 for RCAL and 1.48 for SIMEX) for approach B, which indicates a greater overall precision in approach A.

**Figure 2:**
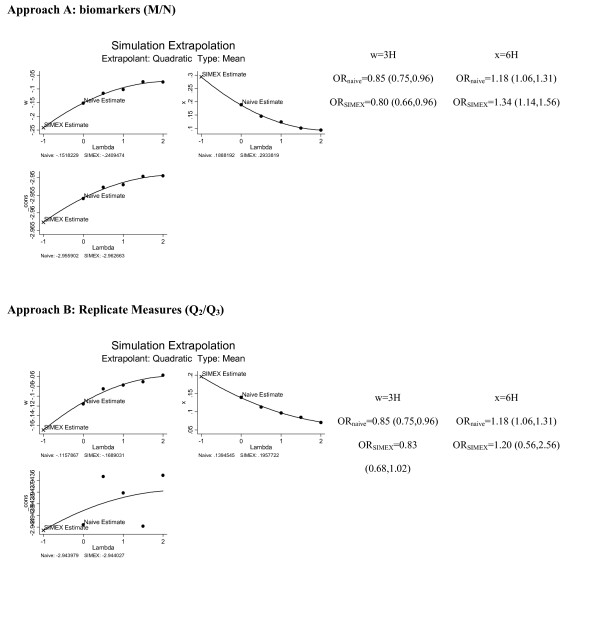
Simulation Extrapolation (SIMEX) plot of corrected coefficients for model with 3H as the exposure and Word Fluency Test (WFT) decline as the outcome: two approaches; ARIC (1987–98)^1^. w: *3H*; x: *6H*; _cons: intercept in the model: Logit(Y = 1) = _cons + *β*_naive1_Q_1(3H) _+ *β*_naive2_Q_1(6H) _where Y = decline in Word Fluency Test (WFT) based on the RCI < -1.645 criterion. Lambda: scale factors used to add error to the error-prone variable Q_1_. Error variance (needed for the SIMEX procedure) is estimated internally using Approach A: {Q_1_, M/N} or Approach B: {Q_1_, Q_2_/Q_3_}.

To illustrate graphically the SIMEX procedure that allows for correction of the point estimate of effect (*β*_1 _= Log_e_OR), Figure 2 shows a SIMEX plot of the association between *3H *(z-score) and odds of decline in WFT corrected using both approaches A (concentration biomarkers M and N) and B (replicate measures of Q_1_: Q_2 _and/or Q_3_). In both cases, *6H *was included as an error prone confounder and the effect of measurement error on its associated regression coefficient is also presented. It is clear that while approach A revealed an underestimation of the protective effect (O R< 1; *β*_1 _= Log_e_(OR) < 0, assuming the true value of 1) of *3H *on WFT decline by an absolute value of 0.09 between the naïve and the SIMEX coefficients, approach B had a discrepancy of no more than 0.05. The same difference in underestimation of adverse effect (OR > 1; *β*_1 _= Log_e_(OR) > 0) is noted for *6H*.

## Discussion

Our main findings suggested that estimates were appreciably less precise for internal repeat measurements when compared to external validation with biomarkers. However, they gave more conservative results regarding the association between fatty acid groups and ratio with cognitive decline in WFT. For instance, based on biomarker/RCAL results, one could conclude that a 1 SD increase in 3H (n-3 highly unsaturated fatty acids) may reduce the odds of decline by as much as 72% or as little as 10%, with an average of 27%. While the point estimate for the repeat measures approach still indicated a protective effect of 3H, both RCAL and SIMEX analyses resulted in broad confidence intervals that crossed the null value of 1. These findings can be contrasted with the study by Duffy and colleagues [5], who found that repeat measurements actually lead to a better precision when compared to external validation. This finding may be caused by an artifact of sample size difference between *Q*_2 _(n = 634) and *Q*_1 _(n = 7,814). However, it is important to note that the main study population differed significantly from the sub-study with external validation data (i.e. biomarkers). In contrast, demographic and lifestyle differences were not as striking for the repeat measurements sub-study, probably due to the relative preservation of racial and ethnic diversity in this sub-group. Hence, transportability of attenuation factors from the validation sub-study is more questionable for the subgroup with available biomarker data (MN whites, n = 2,251). In addition, although loss of precision is an issue, it may also be viewed by some as yielding more conservative estimates of effect particularly if this loss is not substantial, as is the case for SIMEX. Hence, we recommend using SIMEX rather than RCAL whether the choice is to use biomarker measures results (Approach A) or replicate measures (Approach B). As expected, while RCAL gave less precise estimates, however, it corrected for a larger amount of bias in odds ratios, particularly for Approach A.

The present study is also one of the very few attempts to estimate an attenuation factor for essential fatty acids as exposures that can be used subsequently by other researchers for the purpose of correcting for measurement error in bivariate generalized linear models. The approach used was similar to previous research [23-25]. While this article focused on RCAL and SIMEX, other measurement error models utilize the measurement error variance matrix (Σ^uu
 MathType@MTEF@5@5@+=feaafiart1ev1aaatCvAUfKttLearuWrP9MDH5MBPbIqV92AaeXatLxBI9gBaebbnrfifHhDYfgasaacH8akY=wiFfYdH8Gipec8Eeeu0xXdbba9frFj0=OqFfea0dXdd9vqai=hGuQ8kuc9pgc9s8qqaq=dirpe0xb9q8qiLsFr0=vr0=vr0dc8meaabaqaciaacaGaaeqabaqabeGadaaakeaacuqHJoWugaqcamaaBaaaleaacqWG1bqDcqWG1bqDaeqaaaaa@3152@), including methods with instrumental variables and maximum-likelihood methods [26]. Aside from cognitive decline, health outcomes that have traditionally been of interest in relation to essential fatty acids and the balance between them include coronary heart disease [27,28], stroke [29,30], type II diabetes [31], breast and prostate cancer [32,33], depression [34,35], a hypercoagulable profile [36,37] and COPD [38].

One major implication to measurement error, as stated earlier, is loss of statistical power to detect an exposure-disease association. In fact, the sample size required to detect a specific odds ratio (e.g. OR = 2) is inflated proportionally to the inverse squared attenuation factor. For instance, if the true *λ*_j _was 0.2, the sample size, calculated by assuming that *λ*_j _is equal to 0.4, should be multiplied by 0.4^2^/0.2^2 ^= 4 to achieve the same nominal power [39]. It is worth noting that because latent variable T (true intake of fatty acids as % of energy intake) is on a z-score standardized scale, the use of the attenuation factor would lead to a calibrated standardized regression model. For a logistic model, an odds ratio is interpreted as increase or decrease in odds of disease with every SD increase in the continuous z-scored exposure.

Some of the main limitations of this study include the lack of a reference method that is known to be more reliable than FFQs in the ARIC study (e.g. multiple 24-hour recalls or food records). However, because of correlated errors between self-report methods, the use of biomarkers has often been cited as a more adequate means to assess the extent of measurement error in a test instrument. Another drawback is the fact that plasma levels of fatty acids in both fractions studied constitute a short-term measure of intake although they have been shown to correlate well with long-term intake [40]. In addition, the lack of certainty as to the nature of the relationship between the biomarkers considered and the intake variables and the potential interaction of these dietary exposures with other nutritional, environmental and genetic factors constitutes a major challenge for interpretation. For this reason, and using structural equations modeling, estimation of measurement error in FFQ derived nutrients took into consideration two approaches, by including repeat measurements at visit 2 and 3 of the FFQ in one and two concentration biomarkers with assumed linear relationship with intake in another. Finally, although there has been evidence of correlation between intake of fatty acids and their levels in the substrates considered in our study, such a correlation does not necessarily render these biomarkers an adequate reflection of long-term fatty acid intake. In fact, the only substrate that has been shown to work as a gold standard is adipose tissue. However, because of the elevated cost and invasiveness of the procedure, studies using adipose tissue fatty acid concentration as an intake biomarker were often of limited sample size and hence correlations obtained had insufficient levels of precision [41,42]. Another potentially adequate biomarker that was often used to validate medium-term intake of fatty acids is erythrocyte membrane concentration [43,44].

## Conclusion

Future endeavors to correct for error should make use of structural equations modeling and include as many concentration biomarkers as is available along with other self-reported or biomarker-based reference methods of dietary assessment. However, the choice of biomarkers and interpretation of their variability must be made as to account for biochemical and physiological interactions between dietary, environmental and genetic factors. Moreover, one must be cautious of coupled errors between biological markers and must take into account these correlations when specifying the structural model. Finally, because structural equations modeling makes a strong assumption about joint multivariate normality, often not present, it is crucial for future studies to use newly developed methodologies which appear to be more flexible in many ways [45].

## Methods

### Study subjects

ARIC is a prospective cohort study which aimed at investigating the etiology of atherosclerosis and its clinical sequelae and the longitudinal impact of variation in cardiovascular risk factors, medical care, and disease by race, sex, place, and time. In each of four US communities – Forsyth County (NC), Jackson (MS), suburbs of Minneapolis (MN), and Washington County (MD) – 4,000 adults aged 45–64 years were examined four times, three years apart (visits 1 through 4). Three out of the four cohorts represented the ethnic mix of their communities, while at Jackson, MS, only African American residents were recruited [46]. Out of the total sample examined at baseline (N = 15,792) we restricted these analyses to 11,557 individuals aged 50 years or older at baseline. Eligibility for these analyses further required complete data on cognitive functioning at visits 2 (1990–92) and 4 (1996–98) and also complete dietary intake at visit 1 (1987–89), which yielded n = 7,814 men and women. Of these, plasma fatty acid data at visit 1 was available on a sub-set of the Minneapolis cohort, MN (n = 2,251). Additionally, repeat measures using the same FFQ were conducted among 657 at visit 2 and 7,482 at visit 3 of the 7,814 eligible subjects who had exposure data at baseline and complete outcome assessment. Repeat measures on both visits 2 and 3 were available for 634 of the eligible subjects.

### Outcome assessment

Measures of cognitive functioning were made for visits 2 and 4 of the ARIC study. In our present study, we focus on decline in Word Fluency Test (WFT). This test requires subjects to record as many words as possible using the initial letters F, A and S and to list these words, the subject is given only 60 seconds per letter. The total score corresponds to the total number of words generated during these three trials. The test is particularly sensitive to linguistic impairment [47,48] and early mental decline in older persons [49]. It is also a sensitive marker of damage in the left lateral frontal lobe [47,48]. The immediate test-retest correlation coefficient based on an alternate test form has been found to be high (r = 0.82)[50].

Cutoff points were determined for decline in cognitive status WFT test using the Reliable Change Index (RCI) method in order to correct for measurement error and practice effects [51]. RCI is defined as ((X_2_-X_1_)-(M_2_-M_1_))/S.D., where X_1 _is the individual's score at baseline, X_2 _the individual's score at follow-up, M_1 _and M_2 _are the group mean pretest and follow-up scores respectively, and S.D. the observed standard deviation of the difference scores. Scoring below an RCI of -1.645 was regarded as a "statistically reliable" deterioration in the test scores.

### Error-prone dietary exposure

Usual dietary intake was estimated from an interviewer-administered 61-item semi-quantitative food frequency questionnaire (FFQ) previously developed and validated by W. Willet and colleagues against multiple food records among a sub-sample of the Nurse's Health Study cohort [52].

In our study, dietary intake of essential fatty acids and their elongated and desaturated products were expressed as percent of total energy intake and grouped under four main categories, as suggested by Lands and colleagues [53,54]: **(3P) **n-3 C_18 _polyunsaturated fatty acids: 18:3+18:4n-3 **(6P) **n-6 C_18 _polyunsaturated fatty acids: 18:2+18:3n-6 **(3H) **n-3 C_20 _and C_22 _highly unsaturated fatty acids (HUFAs): 20:5+22:5+22:6n-3 and **(6H) **n-6 HUFAs: 20:3+20:4+22:4+22:5n-6. Sums of fatty acid intake as percent of energy included (3) = (3P)+(3H) and (6) = (6P)+(6H). Ratios of interest included (3P)/(6P), (3H)/(6H) and (3P+3H)/(6P+6H) also denoted as 3/6. In multivariate models, all exposure variables were standardized by subtracting each observation from the variable mean and dividing the difference by the standard deviation. Adjustment was made for the other fatty acid variables when appropriate, and total energy intake was considered as a potential confounder to emulate a multivariate nutrient density model [55]. While other energy adjustment methods were possible, the latter was considered as more amenable to public health implications.

### Concentration biomarkers

Twelve-hour fasting blood was collected according to the ARIC study wide protocol. The Minneapolis field center conducted fatty acid analysis in plasma phospholipid and cholesteryl ester fractions for visit 1 blood specimens (1987–89) among the white segment of the study population in that center. The procedure is described in detail elsewhere [38]. The identity of 28 fatty acid peaks were revealed by gas chromatography by comparing each peak's retention time to the retention times of fatty acids in synthetic standards of known compositions. The relative amount of each fatty acid (as a percent of all fatty acids) could be calculated by integrating the area under the peak and dividing the result by the total area for all fatty acids and multiplying by 100. To minimize transcription errors, data from the chromatogram was transferred electronically to a computer for analysis. Two concentration biomarkers, consisting of the plasma phospholipids and cholesteryl ester level of fatty acids in each of the groups described above, were used to assess measurement error in the FFQ and correct for that error.

### Repeat FFQ measures

Dietary intake was assessed among the surviving ARIC sample at visit 3 (1992–94), using the same FFQ that was administered at baseline. At visit 2 (i.e. 1990–92), a sub-sample of ARIC (around 10% of the original sample) was asked to repeat the FFQ, unlike visits 1 and 3 in which the whole ARIC sample was covered. As stated earlier, of our eligible subset with baseline data on exposure and complete outcome data (n = 7,814), 657 had data on visit 2 exposure, 7,482 had complete data at visit 3, while 634 had both.

### Covariates

Most covariates considered as potential confounders were measured at visits 1 or 2. These included sociodemographics (age, gender, ethnicity, education), genetic (Apo E *ε*4 carrier status), behavioral (smoking, alcohol and caffeine consumption and physical activity), nutritional (body mass index, intake of antioxidants and other micronutrients mainly Vitamins B_6_, B_12 _and folate). All these were previously shown to be independently predictive of the outcome and associated with our exposure. The distributions of these covariates within each sub-sample considered are presented in Table 4.

**Table 4:** Baseline characteristics for subgroups (data on Q_1_, additional data on biomarkers M/N, and on replicates Q_2 _and Q_3_); ARIC, 1987

	**Q_1 _**(n = 7,814)		**Q_1_, M and N **(n = 2,251)		**Q_1_, Q_2 _and Q_3 _**(n = 634)	
		
	n	%	n	%	n	%
White	6,387	81.5	2,251	100*	501	79.0
Female	4,269	54.6	1,141	50.7*	293	46.2*
Age(years)						
50–54	2,904	37.2	896	39.8*	179	28.2*
55–59	2,619	33.5	743	33.0	247	38.9
60+	2,291	29.3	612	27.2	208	32.8
Education						
Less than High school	1,580	20.2	150	6.7*	141	22.3
High school graduate	2,658	34.1	814	36.2	189	29.9
More than High school	3,567	45.7	1,286	57.2	303	47.9
Apo E *ε*4 allele	2,249	30.0	614	28.8	164	27.0
Body Mass Index (kg per m^2^)						
<25.00	2,596	33.2	760	33.8*	220	34.7
25.0–29.9	3,220	41.2	962	42.8	274	43.2
≥ 30	1,995	25.5	528	23.5	140	22.1
Smoking status						
Current smoker	1,555	19.9	400	17.8*	136	21.4
Former smoker	2,776	35.5	941	41.8	227	35.8
Never smoked	3,478	44.5	909	40.4	271	42.7
	Mean	(SD)	Mean	(SD)	Mean	(SD)
Alcohol (g/d)	5.9	(12.7)	8.1	(13.5)*	5.6	(11.2)
Caffeine (mg/day)	291.04	(290.82)	348.08	(325.93)*	313.36	(297.42)*
Physical activity scale	7.06	(1.39)	7.33	(1.33)*	7.10	(1.48)
Energy intake (kcal/day)	1578	(571)	1581	(559)	1620	(566)
Vitamin A (1000 IUs/day)	9.1	(7.0)	8.6	(6.8)*	9.2	(7.3)
Vitamin B_6 _(mg/day)	1.75	(0.67)	1.74	(0.66)	1.75	(0.68)
Vitamin B_12 _(mcg/day)	7.61	(4.23)	7.06	(3.50)*	7.97	(4.32)*
Vitamin C (mg/day)	122	(81)	113	(70)*	122	(86)
Vitamin E (mg/day)	5.0	(3.1)	4.7	(3.0)*	5.1	(3.4)
Folate (mcg/day)	232.59	(101.18)	218.48	(94.97)*	235.03	(102.66)

*p < 0.05 for hypothesis that the distribution of the categorical variables is independent of the group or that mean in (Q_1_) group is equal to mean in each of the other group for continuous variables.

### Statistical analysis

**(1) *Bivariate scenario: estimation of attenuation factors***. In this part, each essential fatty acid using the two alternative approaches and a structural equations modeling technique. **(2) *Multivariate scenario: regression calibration and Simulation Extrapolation***. In this part, the association between essential fatty acid intake and clinically significant decline in WFT as an outcome was studied and adjusted for measurement error in exposures. We examined changes in 95% CI and point estimates after correction for error using alternative approaches and methods.

#### (1) Bivariate scenario: estimation of attenuation factors

Estimating the attenuation factor associated with the effect of each exposure variable or covariate on an outcome constitutes the first step for measurement error adjustment using regression calibration. In our example, and for attenuation factor estimation, the structural models were considered for each of the two approaches **(Eq. 1 and 2)**.

Approach A

Q1ij=α01Q1j+α11Q1jTij+εQ1ijMij=α0Mj+α1MjTij+εMijNij=α0Nj+α1NjTij+εNij
 MathType@MTEF@5@5@+=feaafiart1ev1aaatCvAUfKttLearuWrP9MDH5MBPbIqV92AaeXatLxBI9gBaebbnrfifHhDYfgasaacH8akY=wiFfYdH8Gipec8Eeeu0xXdbba9frFj0=OqFfea0dXdd9vqai=hGuQ8kuc9pgc9s8qqaq=dirpe0xb9q8qiLsFr0=vr0=vr0dc8meaabaqaciaacaGaaeqabaqabeGadaaakeaafaqaaeWabaaabaGaemyuae1aaSbaaSqaaiabigdaXiabdMgaPjabdQgaQbqabaaccaGccqWF9aqpiiGacqGFXoqydaWgaaWcbaGaeGimaaJaeGymaeJaemyuae1aaSbaaWqaaiabigdaXiabbQgaQbqabaaaleqaaOGae83kaSIae4xSde2aaSbaaSqaaiabbgdaXiabbgdaXiabbgfarnaaBaaameaacqqGXaqmcqqGQbGAaeqaaaWcbeaakiabdsfaunaaBaaaleaacqWGPbqAcqWGQbGAaeqaaOGae83kaSIae4xTdu2aaSbaaSqaaiabbgfarnaaBaaameaacqqGXaqmcqqGPbqAcqqGQbGAaeqaaaWcbeaaaOqaaiabd2eannaaBaaaleaacqWGPbqAcqWGQbGAaeqaaOGae8xpa0Jae4xSde2aaSbaaSqaaiabicdaWiabb2eannaaBaaameaacqqGQbGAaeqaaaWcbeaakiab=TcaRiab+f7aHnaaBaaaleaacqqGXaqmieGacqqFnbqtdaWgaaadbaGaeeOAaOgabeaaaSqabaGccqWGubavdaWgaaWcbaGaemyAaKMaemOAaOgabeaakiab=TcaRiab+v7aLnaaBaaaleaacqqGnbqtdaWgaaadbaGaeeyAaKMaeeOAaOgabeaaaSqabaaakeaacqWGobGtdaWgaaWcbaGaemyAaKMaemOAaOgabeaakiab=1da9iab+f7aHnaaBaaaleaacqaIWaamcqqGobGtdaWgaaadbaGaeeOAaOgabeaaaSqabaGccqWFRaWkcqGFXoqydaWgaaWcbaGaeGymaeJaemOta40aaSbaaWqaaiabbQgaQbqabaaaleqaaOGaemivaq1aaSbaaSqaaiabdMgaPjabdQgaQbqabaGccqWFRaWkcqGF1oqzdaWgaaWcbaGaeeOta40aaSbaaWqaaiabbMgaPjabbQgaQbqabaaaleqaaaaaaaa@857D@

Approach B

Q1ij=α01Q1j+α11Q1jTij+εQ1ijQ2ij=α02Q2j+α12Q2jTij+εQ2ijQ3ij=α03Q3j+α13Q3jTij+εQ3ij
 MathType@MTEF@5@5@+=feaafiart1ev1aaatCvAUfKttLearuWrP9MDH5MBPbIqV92AaeXatLxBI9gBaebbnrfifHhDYfgasaacH8akY=wiFfYdH8Gipec8Eeeu0xXdbba9frFj0=OqFfea0dXdd9vqai=hGuQ8kuc9pgc9s8qqaq=dirpe0xb9q8qiLsFr0=vr0=vr0dc8meaabaqaciaacaGaaeqabaqabeGadaaakeaafaqaaeWabaaabaGaemyuae1aaSbaaSqaaiabigdaXiabdMgaPjabdQgaQbqabaGccqGH9aqpiiGacqWFXoqydaWgaaWcbaGaeGimaaJaeGymaeJaeeyuae1aaSbaaWqaaiabigdaXiabbQgaQbqabaaaleqaaOGaey4kaSIae8xSde2aaSbaaSqaaiabigdaXiabigdaXiabbgfarnaaBaaameaacqaIXaqmcqqGQbGAaeqaaaWcbeaakiabdsfaunaaBaaaleaacqWGPbqAcqWGQbGAaeqaaOGaey4kaSIae8xTdu2aaSbaaSqaaiabdgfarnaaBaaameaacqqGXaqmcqqGPbqAcqqGQbGAaeqaaaWcbeaaaOqaaiabdgfarnaaBaaaleaacqaIYaGmcqWGPbqAcqWGQbGAaeqaaOGaeyypa0Jae8xSde2aaSbaaSqaaiabicdaWiabikdaYiabbgfarnaaBaaameaacqaIYaGmcqqGQbGAaeqaaaWcbeaakiabgUcaRiab=f7aHnaaBaaaleaacqaIXaqmcqaIYaGmcqqGrbqudaWgaaadbaGaeGOmaiJaeeOAaOgabeaaaSqabaGccqWGubavdaWgaaWcbaGaemyAaKMaemOAaOgabeaakiabgUcaRiab=v7aLnaaBaaaleaacqqGrbqudaWgaaadbaGaeGOmaiJaeeyAaKMaeeOAaOgabeaaaSqabaaakeaacqWGrbqudaWgaaWcbaGaeG4mamJaemyAaKMaemOAaOgabeaakiabg2da9iab=f7aHnaaBaaaleaacqaIWaamcqaIZaWmcqqGrbqudaWgaaadbaGaeG4mamJaeeOAaOgabeaaaSqabaGccqGHRaWkcqWFXoqydaWgaaWcbaGaeGymaeJaeG4mamJaeeyuae1aaSbaaWqaaiabiodaZiabbQgaQbqabaaaleqaaOGaemivaq1aaSbaaSqaaiabdMgaPjabdQgaQbqabaGccqGHRaWkcqWF1oqzdaWgaaWcbaGaeeyuae1aaSbaaWqaaiabiodaZiabbMgaPjabbQgaQbqabaaaleqaaaaaaaa@916A@

This set of equations allowed us to estimate the attenuation factor *λ*_j _with its approximate SE, using the delta method (**Eq. 3**).

λj=Cov(Q1,T)/Var(Q1)=α11Q1jα11Q1j2+σεQ1j2/σT2=Corr(Q1,T)=α11Q1j
 MathType@MTEF@5@5@+=feaafiart1ev1aaatCvAUfKttLearuWrP9MDH5MBPbIqV92AaeXatLxBI9gBaebbnrfifHhDYfgasaacH8akY=wiFfYdH8Gipec8Eeeu0xXdbba9frFj0=OqFfea0dXdd9vqai=hGuQ8kuc9pgc9s8qqaq=dirpe0xb9q8qiLsFr0=vr0=vr0dc8meaabaqaciaacaGaaeqabaqabeGadaaakeaaiiGacqWF7oaBdaWgaaWcbaGaemOAaOgabeaakiabg2da9iabboeadjabb+gaVjabbAha2jabcIcaOiabdgfarnaaBaaaleaacqaIXaqmaeqaaOGaeiilaWIaemivaqLaeiykaKIaei4la8IaeeOvayLaeeyyaeMaeeOCaiNaeiikaGIaemyuae1aaSbaaSqaaiabigdaXaqabaGccqGGPaqkcqGH9aqpdaWcaaqaaiab=f7aHnaaBaaaleaacqaIXaqmcqaIXaqmcqWGrbqudaWgaaadbaGaeGymaeJaemOAaOgabeaaaSqabaaakeaacqWFXoqydaqhaaWcbaGaeGymaeJaeGymaeJaemyuae1aaSbaaWqaaiabigdaXiabdQgaQbqabaaaleaacqaIYaGmaaGccqGHRaWkcqWFdpWCdaqhaaWcbaGae8xTdu2aaSbaaWqaaiabdgfarjabigdaXmaaBaaabaGaemOAaOgabeaaaeqaaaWcbaGaeGOmaidaaOGaei4la8Iae83Wdm3aa0baaSqaaiabdsfaubqaaiabikdaYaaaaaGccqGH9aqpcqqGdbWqcqqGVbWBcqqGYbGCcqqGYbGCcqGGOaakcqWGrbqudaWgaaWcbaGaeGymaedabeaakiabcYcaSiabdsfaujabcMcaPiabg2da9iab=f7aHnaaBaaaleaacqaIXaqmcqaIXaqmcqWGrbqudaWgaaadbaGaeGymaeJaemOAaOgabeaaaSqabaaaaa@778A@

when Var(Q_1_) = Var(T) = 1. This is the case when all measured variables (Q_k_, M, N) are standardized z-scores.

The lower the attenuation factor, the higher the measurement error in the error-prone exposure variable. *λ*_j _= 1

if there is no measurement error or Q_1 _= T.

Assuming non-differential misclassification, dietary measurement error often attenuates diet-disease relationship and thus biases the effect towards the null. The amount by which the association is biased can be estimated by the attenuation factor [25]. According to Rosner and colleagues [56], if a primary regression model **(Eq. 4.1) **with a response variable *Y *contains only one error-prone explanatory variable *Q*_*j *_(bivariate scenario) correction for attenuation, consists simply of dividing the regression coefficient *β*_(naïve, j) _of that variable *Q*_*j *_by the attenuation factor *λ*_j _**(Eq. 4.2)**. The variance estimates for the corrected regression coefficient of effect as well as 95 percent CI are estimated using Eqs. **4.3.1, 4.3.2 **and **4.4**. *Q*_1*ij *_and biomarkers *M*_*ij *_and *N*_*ij *_are entered into the model as z-scored variables. The same applies to the repeat measures *Q*_2*ij *_and *Q*_3*ij. *_Note that *Q*_1*ij *_is the manifest variable denoting visit 1 dietary exposures, which are the test exposures of interest (n = 7,814).

E(Y|Qj)=β^0+β^(naïve, j)Qj
 MathType@MTEF@5@5@+=feaafiart1ev1aaatCvAUfKttLearuWrP9MDH5MBPbIqV92AaeXatLxBI9gBaebbnrfifHhDYfgasaacH8akY=wiFfYdH8Gipec8Eeeu0xXdbba9frFj0=OqFfea0dXdd9vqai=hGuQ8kuc9pgc9s8qqaq=dirpe0xb9q8qiLsFr0=vr0=vr0dc8meaabaqaciaacaGaaeqabaqabeGadaaakeaacqqGfbqrcqqGOaakcqWGzbqwcqqG8baFcqWGrbqudaWgaaWcbaGaemOAaOgabeaakiabbMcaPGGaaiab=1da9GGaciqb+j7aIzaajaWaaSbaaSqaaiabbcdaWaqabaGccqWFRaWkcuGFYoGygaqcamaaBaaaleaacqqGOaakcqqGUbGBcqqGHbqycqqGVdW7cqqG2bGDcqqGLbqzcqqGSaalcqqGGaaicqqGQbGAcqqGPaqkaeqaaOGaemyuae1aaSbaaSqaaiabdQgaQbqabaaaaa@4AB8@

β^(RC, j)=β^(naïve, j) / λ^j
 MathType@MTEF@5@5@+=feaafiart1ev1aaatCvAUfKttLearuWrP9MDH5MBPbIqV92AaeXatLxBI9gBaebbnrfifHhDYfgasaacH8akY=wiFfYdH8Gipec8Eeeu0xXdbba9frFj0=OqFfea0dXdd9vqai=hGuQ8kuc9pgc9s8qqaq=dirpe0xb9q8qiLsFr0=vr0=vr0dc8meaabaqaciaacaGaaeqabaqabeGadaaakeaaiiGacuWFYoGygaqcamaaBaaaleaacqqGOaakcqqGsbGucqqGdbWqcqqGSaalcqqGGaaicqqGQbGAcqqGPaqkaeqaaGGaaOGae4xpa0Jaf8NSdiMbaKaadaWgaaWcbaGaeeikaGIaeeOBa4MaeeyyaeMaee47a8UaeeODayNaeeyzauMaeeilaWIaeeiiaaIaeeOAaOMaeeykaKcabeaakiabbccaGiabb+caViabbccaGiqb=T7aSzaajaWaaSbaaSqaaiabbQgaQbqabaaaaa@4AAC@

Var(β^(RC, j))=Var(β^(naïve, j))/λ^j2+β^(naïve, j)2Var(λ^j)/λ^j4 
 MathType@MTEF@5@5@+=feaafiart1ev1aaatCvAUfKttLearuWrP9MDH5MBPbIqV92AaeXatLxBI9gBamXvP5wqSXMqHnxAJn0BKvguHDwzZbqegyvzYrwyUfgarqqtubsr4rNCHbGeaGqiA8vkIkVAFgIELiFeLkFeLk=iY=Hhbbf9v8qqaqFr0xc9pk0xbba9q8WqFfeaY=biLkVcLq=JHqVepeea0=as0db9vqpepesP0xe9Fve9Fve9GapdbaqaaeGacaGaaiaabeqaamqadiabaaGceaqabeaacqqGwbGvcqqGHbqycqqGYbGCcqqGOaakiiGacuWFYoGygaqcamaaBaaaleaacqqGOaakcqqGsbGucqqGdbWqcqqGSaalcqqGGaaicqqGQbGAcqqGPaqkaeqaaOGaeeykaKcccaGae4xpa0dabaGaeeOvayLaeeyyaeMaeeOCaiNaeeikaGIaf8NSdiMbaKaadaWgaaWcbaGaeeikaGIaeeOBa4MaeeyyaeMaee47a8UaeeODayNaeeyzauMaeeilaWIaeeiiaaIaeeOAaOMaeeykaKcabeaakiabbMcaPiabb+caViqb=T7aSzaajaWaa0baaSqaaiabdQgaQbqaaiabikdaYaaakiab+TcaRiqb=j7aIzaajaWaa0baaSqaaiabbIcaOiabb6gaUjabbggaHjabb+oaVlabbAha2jabbwgaLjabbYcaSiabbccaGiabbQgaQjabbMcaPaqaaiabbkdaYaaakiabbAfawjabbggaHjabbkhaYjabbIcaOiqb=T7aSzaajaWaaSbaaSqaaiabbQgaQbqabaGccqqGPaqkcqqGVaWlcuWF7oaBgaqcamaaDaaaleaacqWGQbGAaeaacqaI0aanaaGccqqGGaaiaaaa@849B@

SE(β^(RC, j))=Var(β^(RC,j))
 MathType@MTEF@5@5@+=feaafiart1ev1aaatCvAUfKttLearuWrP9MDH5MBPbIqV92AaeXatLxBI9gBaebbnrfifHhDYfgasaacH8akY=wiFfYdH8Gipec8Eeeu0xXdbba9frFj0=OqFfea0dXdd9vqai=hGuQ8kuc9pgc9s8qqaq=dirpe0xb9q8qiLsFr0=vr0=vr0dc8meaabaqaciaacaGaaeqabaqabeGadaaakeaacqqGtbWucqqGfbqrcqqGOaakiiGacuWFYoGygaqcamaaBaaaleaacqqGOaakcqqGsbGucqqGdbWqcqqGSaalcqqGGaaicqqGQbGAcqqGPaqkaeqaaOGaeeykaKcccaGae4xpa0ZaaOaaaeaacqqGwbGvcqqGHbqycqqGYbGCcqqGOaakcuWFYoGygaqcamaaBaaaleaacqqGOaakcqqGsbGucqqGdbWqcqqGSaalcqqGQbGAcqqGPaqkaeqaaOGaeeykaKcaleqaaaaa@482F@

95 percent CI β(RC, j) ≡β^(RC, j)±1.96×SE(β^(RC, j))
 MathType@MTEF@5@5@+=feaafiart1ev1aaatCvAUfKttLearuWrP9MDH5MBPbIqV92AaeXatLxBI9gBaebbnrfifHhDYfgasaacH8akY=wiFfYdH8Gipec8Eeeu0xXdbba9frFj0=OqFfea0dXdd9vqai=hGuQ8kuc9pgc9s8qqaq=dirpe0xb9q8qiLsFr0=vr0=vr0dc8meaabaqaciaacaGaaeqabaqabeGadaaakqaabeqaaiabbMda5iabbwda1iabbccaGiabbchaWjabbwgaLjabbkhaYjabbogaJjabbwgaLjabb6gaUjabbsha0jabbccaGiabboeadjabbMeajjabbccaGGGaciab=j7aInaaBaaaleaacqqGOaakcqqGsbGucqqGdbWqcqqGSaalcqqGGaaicqqGQbGAcqqGPaqkcqqGGaaiaeqaaOGaeyyyIOlabaGaf8NSdiMbaKaadaWgaaWcbaGaeeikaGIaeeOuaiLaee4qamKaeeilaWIaeeiiaaIaeeOAaOMaeeykaKcabeaaiiaakiab+flaXkabbgdaXiabb6caUiabbMda5iabbAda2iab+Dna0kabbofatjabbweafjabbIcaOiqb=j7aIzaajaWaaSbaaSqaaiabbIcaOiabbkfasjabboeadjabbYcaSiabbccaGiabbQgaQjabbMcaPaqabaGccqqGPaqkaaaa@653D@

For logistic regression, the assumptions made are linear homoscedastic regression of T on Q with a normally distributed error term and a rare disease requirement [56]. Subsequently Kuha [57] introduced two key requirements for approximate unbiasedness of *β*_RC _: **(i) ***β*^2^_1 _* *σ*^2 ^product is small; where *σ*^2 ^= Var (*T*|*Q*, *Z*_*k*_); **(ii) **Pr(*Y *= 1|*T*) is small and f(*T*|*Q*) is normal.

In cases where more than one error-prone variable and several perfectly measured variables are introduced into a primary regression model **(5)**, the attenuation factor is no longer the parameter to be used for calibration.

E(Y|Qm,Zn)=β^0+∑m=1pβ^T,naïve,mQm+∑n=1qβ^Z,naïve,nZn
 MathType@MTEF@5@5@+=feaafiart1ev1aaatCvAUfKttLearuWrP9MDH5MBPbIqV92AaeXatLxBI9gBamXvP5wqSXMqHnxAJn0BKvguHDwzZbqegyvzYrwyUfgarqqtubsr4rNCHbGeaGqiA8vkIkVAFgIELiFeLkFeLk=iY=Hhbbf9v8qqaqFr0xc9pk0xbba9q8WqFfeaY=biLkVcLq=JHqVepeea0=as0db9vqpepesP0xe9Fve9Fve9GapdbaqaaeGacaGaaiaabeqaamqadiabaaGcbaGaeeyrauKaeeikaGIaemywaKLaeeiFaWNaemyuae1aaSbaaSqaaiabd2gaTbqabaGccqGGSaalcqWGGaaicqqGAbGwdaWgaaWcbaGaemOBa4gabeaakiabbMcaPGGaaiab=1da9GGaciqb+j7aIzaajaWaaSbaaSqaaiabbcdaWaqabaGccqWFRaWkdaaeWbqaaiqb+j7aIzaajaWaaSbaaSqaaiabbsfaujabbYcaSiabb6gaUjabbggaHjabb+oaVlabbAha2jabbwgaLjabbYcaSiabd2gaTbqabaGccqWGrbqudaWgaaWcbaGaemyBa0gabeaaaeaacqWGTbqBcqGH9aqpcqaIXaqmaeaacqWGWbaCa0GaeyyeIuoakiab=TcaRmaaqahabaGaf4NSdiMbaKaadaWgaaWcbaGaeeOwaOLagiilaWIaeiOBa4MaeiyyaeMaei47a8UaeiODayNaeiyzauMaeiilaWIaemOBa4gabeaakiabbQfaAnaaBaaaleaacqWGUbGBaeqaaaqaaiabd6gaUjabb2da9iabbgdaXaqaaiabbghaXbqdcqGHris5aaaa@7ED7@

#### (2) Multivariate scenario: regression calibration and simulation extrapolation

For the multivariate scenario, we computed odds ratios of clinically significant decline in WFT (RCI < -1.645) with increase in each exposure by 1 SD through a multivariate logistic regression analysis. Control for confounding was accomplished using backward elimination whereby covariates that changed the estimated effect of the exposure by more than 5% were retained in the final model [58]. The parsimonious model which provided a non-confounded estimate of the effect of a fatty acid exposure on the outcome (decline in WFT over a period of six years: a binary variable) was represented as the naïve estimate of effect. Subsequently, regression calibration and simulation extrapolation were conducted on this same parsimonious model, as alternative methods for two approaches (A and B) described earlier.

Under such a multivariate scenario, regression calibration becomes reliant on the variance-covariance matrix of error in the measurement of different error-prone variables *Q*_*j *_(Σ^uu
 MathType@MTEF@5@5@+=feaafiart1ev1aaatCvAUfKttLearuWrP9MDH5MBPbIqV92AaeXatLxBI9gBaebbnrfifHhDYfgasaacH8akY=wiFfYdH8Gipec8Eeeu0xXdbba9frFj0=OqFfea0dXdd9vqai=hGuQ8kuc9pgc9s8qqaq=dirpe0xb9q8qiLsFr0=vr0=vr0dc8meaabaqaciaacaGaaeqabaqabeGadaaakeaacuqHJoWugaqcamaaBaaaleaacqqG1bqDcqqG1bqDaeqaaaaa@314E@) as well as the variance-covariance matrices of the variables themselves. This relationship termed "method of moments" can be summarized in equation **(6) **[26].

(β^Z,RCβ^T,RC)=(ΣZZΣZQΣQZΣQQ−Σ^uu)−1(ΣZZΣZQΣQZΣQQ)(β^Z,naiveβ^T,naive)
 MathType@MTEF@5@5@+=feaafiart1ev1aaatCvAUfKttLearuWrP9MDH5MBPbIqV92AaeXatLxBI9gBaebbnrfifHhDYfgasaacH8akY=wiFfYdH8Gipec8Eeeu0xXdbba9frFj0=OqFfea0dXdd9vqai=hGuQ8kuc9pgc9s8qqaq=dirpe0xb9q8qiLsFr0=vr0=vr0dc8meaabaqaciaacaGaaeqabaqabeGadaaakeaadaqadaqaauaabeqaceaaaeaaiiGacuWFYoGygaqcamaaBaaaleaacqWGAbGwcqGGSaalcqWGsbGucqWGdbWqaeqaaaGcbaGaf8NSdiMbaKaadaWgaaWcbaGaemivaqLaeiilaWIaemOuaiLaem4qameabeaaaaaakiaawIcacaGLPaaacqGH9aqpdaqadaqaauaabeqaciaaaeaacqqHJoWudaWgaaWcbaGaemOwaOLaemOwaOfabeaaaOqaaiabfo6atnaaBaaaleaacqWGAbGwcqWGrbquaeqaaaGcbaGaeu4Odm1aaSbaaSqaaiabdgfarjabdQfaAbqabaaakeaacqqHJoWudaWgaaWcbaGaemyuaeLaemyuaefabeaakiabgkHiTiqbfo6atzaajaWaaSbaaSqaaiabdwha1jabdwha1bqabaaaaaGccaGLOaGaayzkaaWaaWbaaSqabeaacqGHsislcqaIXaqmaaGcdaqadaqaauaabeqaciaaaeaacqqHJoWudaWgaaWcbaGaemOwaOLaemOwaOfabeaaaOqaaiabfo6atnaaBaaaleaacqWGAbGwcqWGrbquaeqaaaGcbaGaeu4Odm1aaSbaaSqaaiabdgfarjabdQfaAbqabaaakeaacqqHJoWudaWgaaWcbaGaemyuaeLaemyuaefabeaaaaaakiaawIcacaGLPaaadaqadaqaauaabeqaceaaaeaacuWFYoGygaqcamaaBaaaleaacqWGAbGwcqGGSaalcqWGUbGBcqWGHbqycqWGPbqAcqWG2bGDcqWGLbqzaeqaaaGcbaGaf8NSdiMbaKaadaWgaaWcbaGaemivaqLaeiilaWIaemOBa4MaemyyaeMaemyAaKMaemODayNaemyzaugabeaaaaaakiaawIcacaGLPaaaaaa@7EDF@

The detailed explanations of each moment is laid out in equations **7.1 **through **7.5 **[59], and table 5.

**Table 5:** Example for equation 7

**Obs. *i***	**Error-prone var. (Q_1 _= W_1_)**	**Replicate (Q_2 _= W_2_) (M = W_2_)**	**Replicate 2...k (Q_3 _= W_3_) (N = W_3_)**	**Non-error prone var. (Z)**	**Y**
1	1.02	1.05	1.10	55	0
2	0.80	Missing	0.65	60	1
3	1.50	Missing	Missing	62	0
...	...	...	...	...	...
n	n_Q1 _= 7,814	n_M _= 2,251	n_N _= 2,251	n_Z _= n_Y _= 7,814	
		n_Q2 _= 657	n_Q3 _= 7,482		

**Example**: FFQ visit 1 z-score for fatty acid exposures (Q1) are error-prone variables and have at most two replicates: 1 ≤ k ≤ 3 for each observation. Z (e.g. age) is available for all observations and is not error-prone.

Σ^uu=1∑i=1n(ki−1)∑i=1n∑j=1ki(Wij−W¯i.)(Wij−W¯i.)T
 MathType@MTEF@5@5@+=feaafiart1ev1aaatCvAUfKttLearuWrP9MDH5MBPbIqV92AaeXatLxBI9gBaebbnrfifHhDYfgasaacH8akY=wiFfYdH8Gipec8Eeeu0xXdbba9frFj0=OqFfea0dXdd9vqai=hGuQ8kuc9pgc9s8qqaq=dirpe0xb9q8qiLsFr0=vr0=vr0dc8meaabaqaciaacaGaaeqabaqabeGadaaakeaacuqHJoWugaqcamaaBaaaleaacqWG1bqDcqWG1bqDaeqaaOGaeyypa0ZaaSaaaeaacqaIXaqmaeaadaaeWbqaaiabcIcaOiabdUgaRnaaBaaaleaacqWGPbqAaeqaaOGaeyOeI0IaeGymaeJaeiykaKcaleaacqWGPbqAcqGH9aqpcqaIXaqmaeaacqWGUbGBa0GaeyyeIuoaaaGcdaaeWbqaamaaqahabaGaeiikaGIaem4vaC1aaSbaaSqaaiabdMgaPjabdQgaQbqabaGccqGHsislcuWGxbWvgaqeamaaBaaaleaacqWGPbqAcqGGUaGlaeqaaOGaeiykaKcaleaacqWGQbGAcqGH9aqpcqaIXaqmaeaacqWGRbWAdaWgaaadbaGaemyAaKgabeaaa0GaeyyeIuoaaSqaaiabdMgaPjabg2da9iabigdaXaqaaiabd6gaUbqdcqGHris5aOGaeiikaGIaem4vaC1aaSbaaSqaaiabdMgaPjabdQgaQbqabaGccqGHsislcuWGxbWvgaqeamaaBaaaleaacqWGPbqAcqGGUaGlaeqaaOGaeiykaKYaaWbaaSqabeaacqWGubavaaaaaa@66CF@

k replicates for each individual i. W = {Q_1_, Q_2_/Q_3_} or {Q_1_, M/N}.

Σ^ZZ=1n−1∑i=1n(Zi−Z¯.)(Zi−Z¯i.)T
 MathType@MTEF@5@5@+=feaafiart1ev1aaatCvAUfKttLearuWrP9MDH5MBPbIqV92AaeXatLxBI9gBaebbnrfifHhDYfgasaacH8akY=wiFfYdH8Gipec8Eeeu0xXdbba9frFj0=OqFfea0dXdd9vqai=hGuQ8kuc9pgc9s8qqaq=dirpe0xb9q8qiLsFr0=vr0=vr0dc8meaabaqaciaacaGaaeqabaqabeGadaaakeaacuqHJoWugaqcamaaBaaaleaacqWGAbGwcqWGAbGwaeqaaOGaeyypa0ZaaSaaaeaacqaIXaqmaeaacqWGUbGBcqGHsislcqaIXaqmaaWaaabCaeaacqGGOaakcqWGAbGwdaWgaaWcbaGaemyAaKgabeaakiabgkHiTiqbdQfaAzaaraWaaSbaaSqaaiabc6caUaqabaGccqGGPaqkaSqaaiabdMgaPjabg2da9iabigdaXaqaaiabd6gaUbqdcqGHris5aOGaeiikaGIaemOwaO1aaSbaaSqaaiabdMgaPbqabaGccqGHsislcuWGAbGwgaqeamaaBaaaleaacqWGPbqAcqGGUaGlaeqaaOGaeiykaKYaaWbaaSqabeaacqWGubavaaaaaa@4FAB@

Σ^QZ=1ν∑i=1nki(W¯i.−μ^W)(Zi−Z¯i.)T
 MathType@MTEF@5@5@+=feaafiart1ev1aaatCvAUfKttLearuWrP9MDH5MBPbIqV92AaeXatLxBI9gBaebbnrfifHhDYfgasaacH8akY=wiFfYdH8Gipec8Eeeu0xXdbba9frFj0=OqFfea0dXdd9vqai=hGuQ8kuc9pgc9s8qqaq=dirpe0xb9q8qiLsFr0=vr0=vr0dc8meaabaqaciaacaGaaeqabaqabeGadaaakeaacuqHJoWugaqcamaaBaaaleaacqWGrbqucqWGAbGwaeqaaOGaeyypa0ZaaSaaaeaacqaIXaqmaeaaiiGacqWF9oGBaaWaaabCaeaacqWGRbWAdaWgaaWcbaGaemyAaKgabeaakiabcIcaOiqbdEfaxzaaraWaaSbaaSqaaiabdMgaPjabc6caUaqabaGccqGHsislcuWF8oqBgaqcamaaBaaaleaacqWGxbWvaeqaaOGaeiykaKcaleaacqWGPbqAcqGH9aqpcqaIXaqmaeaacqWGUbGBa0GaeyyeIuoakiabcIcaOiabdQfaAnaaBaaaleaacqWGPbqAaeqaaOGaeyOeI0IafmOwaOLbaebadaWgaaWcbaGaemyAaKMaeiOla4cabeaakiabcMcaPmaaCaaaleqabaGaemivaqfaaaaa@52BB@

Σ^QQ=1ν{∑i=1nki(W¯i.−μ^W)(W¯i.−μ^W)T}−n−1νΣ^uu
 MathType@MTEF@5@5@+=feaafiart1ev1aaatCvAUfKttLearuWrP9MDH5MBPbIqV92AaeXatLxBI9gBaebbnrfifHhDYfgasaacH8akY=wiFfYdH8Gipec8Eeeu0xXdbba9frFj0=OqFfea0dXdd9vqai=hGuQ8kuc9pgc9s8qqaq=dirpe0xb9q8qiLsFr0=vr0=vr0dc8meaabaqaciaacaGaaeqabaqabeGadaaakqaabeqaaiqbfo6atzaajaWaaSbaaSqaaiabdgfarjabdgfarbqabaGccqGH9aqpdaWcaaqaaiabigdaXaqaaGGaciab=17aUbaadaGadaqaamaaqahabaGaem4AaS2aaSbaaSqaaiabdMgaPbqabaGccqGGOaakcuWGxbWvgaqeamaaBaaaleaacqWGPbqAcqGGUaGlaeqaaOGaeyOeI0Iaf8hVd0MbaKaadaWgaaWcbaGaem4vaCfabeaakiabcMcaPaWcbaGaemyAaKMaeyypa0JaeGymaedabaGaemOBa4ganiabggHiLdGccqGGOaakcuWGxbWvgaqeamaaBaaaleaacqWGPbqAcqGGUaGlaeqaaOGaeyOeI0Iaf8hVd0MbaKaadaWgaaWcbaGaem4vaCfabeaakiabcMcaPmaaCaaaleqabaGaemivaqfaaaGccaGL7bGaayzFaaaabaGaeyOeI0YaaSaaaeaacqWGUbGBcqGHsislcqaIXaqmaeaacqWF9oGBaaGafu4OdmLbaKaadaWgaaWcbaGaemyDauNaemyDauhabeaaaaaa@5FDD@

ν=∑iki−∑iki2/∑iki
 MathType@MTEF@5@5@+=feaafiart1ev1aaatCvAUfKttLearuWrP9MDH5MBPbIqV92AaeXatLxBI9gBaebbnrfifHhDYfgasaacH8akY=wiFfYdH8Gipec8Eeeu0xXdbba9frFj0=OqFfea0dXdd9vqai=hGuQ8kuc9pgc9s8qqaq=dirpe0xb9q8qiLsFr0=vr0=vr0dc8meaabaqaciaacaGaaeqabaqabeGadaaakeaaiiGacqWF9oGBcqGH9aqpdaaeqbqaaiabdUgaRnaaBaaaleaacqWGPbqAaeqaaaqaaiabdMgaPbqab0GaeyyeIuoakiabgkHiTmaaqafabaGaem4AaS2aa0baaSqaaiabdMgaPbqaaiabikdaYaaaaeaacqWGPbqAaeqaniabggHiLdGccqGGVaWldaaeqaqaaiabdUgaRnaaBaaaleaacqWGPbqAaeqaaaqaaiabdMgaPbqab0GaeyyeIuoaaaa@4516@

Alternatively, one can use simulation extrapolation (SIMEX) which also relies on the method of moments with an estimate of Σ^uu
 MathType@MTEF@5@5@+=feaafiart1ev1aaatCvAUfKttLearuWrP9MDH5MBPbIqV92AaeXatLxBI9gBaebbnrfifHhDYfgasaacH8akY=wiFfYdH8Gipec8Eeeu0xXdbba9frFj0=OqFfea0dXdd9vqai=hGuQ8kuc9pgc9s8qqaq=dirpe0xb9q8qiLsFr0=vr0=vr0dc8meaabaqaciaacaGaaeqabaqabeGadaaakeaacuqHJoWugaqcamaaBaaaleaacqqG1bqDcqqG1bqDaeqaaaaa@314E@ or replicate measures of the error-prone variable. This method can be summarized as follows[60]:

**Step 1 **: Simulation step:

• Create additional datasets with increasingly larger amounts of measurement error, after estimating error variance for each error-prone variable (σ^u2
 MathType@MTEF@5@5@+=feaafiart1ev1aaatCvAUfKttLearuWrP9MDH5MBPbIqV92AaeXatLxBI9gBaebbnrfifHhDYfgasaacH8akY=wiFfYdH8Gipec8Eeeu0xXdbba9frFj0=OqFfea0dXdd9vqai=hGuQ8kuc9pgc9s8qqaq=dirpe0xb9q8qiLsFr0=vr0=vr0dc8meaabaqaciaacaGaaeqabaqabeGadaaakeaaiiGacuWFdpWCgaqcamaaDaaaleaacqWG1bqDaeaacqaIYaGmaaaaaa@3118@):

(1 + *θ*)σ^u2
 MathType@MTEF@5@5@+=feaafiart1ev1aaatCvAUfKttLearuWrP9MDH5MBPbIqV92AaeXatLxBI9gBaebbnrfifHhDYfgasaacH8akY=wiFfYdH8Gipec8Eeeu0xXdbba9frFj0=OqFfea0dXdd9vqai=hGuQ8kuc9pgc9s8qqaq=dirpe0xb9q8qiLsFr0=vr0=vr0dc8meaabaqaciaacaGaaeqabaqabeGadaaakeaaiiGacuWFdpWCgaqcamaaDaaaleaacqWG1bqDaeaacqaIYaGmaaaaaa@3118@ with: *θ *= {0.5,1.0,1.5,2.0}

*Var*_*θ *_(*Q*) = *Var*(*T*) + ( 1 + *θ*)σ^u2
 MathType@MTEF@5@5@+=feaafiart1ev1aaatCvAUfKttLearuWrP9MDH5MBPbIqV92AaeXatLxBI9gBaebbnrfifHhDYfgasaacH8akY=wiFfYdH8Gipec8Eeeu0xXdbba9frFj0=OqFfea0dXdd9vqai=hGuQ8kuc9pgc9s8qqaq=dirpe0xb9q8qiLsFr0=vr0=vr0dc8meaabaqaciaacaGaaeqabaqabeGadaaakeaaiiGacuWFdpWCgaqcamaaDaaaleaacqWG1bqDaeaacqaIYaGmaaaaaa@3118@

• Regression coefficients estimated using method of moments **(Eq. 6–7.5)**

**Step 2**: Extrapolation step:

After plotting each of the estimated coefficients with *θ *= {0.5,1.0,1.5,2.0}on the x-axis, the coefficient *β*_*T*, *SIMEX *_is estimated by extrapolating backwards to a value of *θ *= -1, where covariate Q becomes error-free,

*Var*(*T*) = *Var*_-1_(*Q*) + (1 + -1)σ^u2
 MathType@MTEF@5@5@+=feaafiart1ev1aaatCvAUfKttLearuWrP9MDH5MBPbIqV92AaeXatLxBI9gBaebbnrfifHhDYfgasaacH8akY=wiFfYdH8Gipec8Eeeu0xXdbba9frFj0=OqFfea0dXdd9vqai=hGuQ8kuc9pgc9s8qqaq=dirpe0xb9q8qiLsFr0=vr0=vr0dc8meaabaqaciaacaGaaeqabaqabeGadaaakeaaiiGacuWFdpWCgaqcamaaDaaaleaacqWG1bqDaeaacqaIYaGmaaaaaa@3118@ = *Var*_-1_(*Q*)

**Step 3**: Variance and SE of the regression coefficients for SIMEX point estimates are estimated either with an asymptotic or bootstrap method.

## Competing interests

The author(s) declare that they have no competing interests.

## Authors' contributions

MAB: Conception, literature review, methods, plan of analysis, data analysis, interpretation of results.

JSK: Conception, methods, plan of analysis, literature review, revision of manuscript.

JI: Methods, plan of analysis, revision of manuscript.

JS: Conception, literature review, plan of analysis, revision of manuscript.

GH: Provision of data, plan of analysis, revision of manuscript.

## Pre-publication history

The pre-publication history for this paper can be accessed here:


